# Empowering community health professionals for effective air pollution information communication

**DOI:** 10.1186/s12889-023-17462-1

**Published:** 2023-12-20

**Authors:** Tricia Tan, Cornelia Junghans, Diana Varaden

**Affiliations:** 1 School of Public Health, Imperial College London, London, UK; 2 Department of Primary Care and Public Health, Imperial College London, London, UK; 3 MRC Centre for Environment and Health, Imperial College London, London, UK; 4 NIHR-HPRU Environmental Exposures and Health, School of Public Health, Imperial College London, London, UK

**Keywords:** Community health professionals, Air pollution, Public health advice, Communication strategy

## Abstract

**Background:**

Air pollution remains a significant public health risk, particularly in urban areas. Effective communication strategies remain integral to overall protection by encouraging the adoption of personal air pollution exposure reduction behaviours. This study aims to explore how community health professionals can be empowered to communicate air pollution information and advice to the wider community, to encourage the uptake of desired behaviours in the population.

**Methods:**

The study adopted a qualitative methodology, where four homogenous Focus Group Discussions (FGDs) were held with a range of community health professionals, including Health Care Professionals, Community Health and Wellbeing Workers (CHWWs) and Social Prescribing Link Workers (SPLW). A classical content analysis was conducted with the Structural Empowerment Theory (SET) and Psychological Empowerment Theory (PET) as guiding concepts.

**Results:**

Five key themes were identified: from a structural empowerment perspective: [1] resources and support, [2] knowledge. From a psychological empowerment perspective: [3] confidence as advisor, [4] responsibility as advisor, and [5] residents’ receptiveness to advice. It was concluded that advice should be risk stratified, clear, easy to follow and provide alternatives.

**Conclusion:**

This study identified ways for community health professionals to be empowered by local councils or other organisations in providing advice on air pollution, through the provision of essential structural support and opportunities to enhance their knowledge and confidence in the subject. Implementing recommendations from this study would not only empower community health professionals to advise on air pollution to the wider community but also increase adherence to health advice.

**Supplementary Information:**

The online version contains supplementary material available at 10.1186/s12889-023-17462-1.

## Background

Air pollution remains one of the largest environmental health threats of modern times. In 2019, air pollution emerged as the leading Level 2 risk factor for DALYs among all environmental and occupational risks, contributing to 213 million DALYs and resulting in 6.67 million deaths, as reported by the recent Global Burden of Diseases (GBD) study [1]. A plethora of evidence suggests that air pollution has negative short-term and long-term health implications, including cardiovascular and respiratory complications, heart disease and lung cancer, all of which contribute to pollution-related mortality [2]. Despite these adverse health effects, the majority of urban populations live in areas with air quality that fails to meet the World Health Organization (WHO) guidelines [3].

### Behaviour change communication

Interventions to mitigate the impacts of air pollution are often multi-sectoral and involve collaboration between various stakeholders. Interventions can be transport, urban planning, industrial, agricultural or behavioural focused [4]. Many interventions are regulatory and involve legislation and cooperation between the government and industry. This study focuses on short term behavioural interventions, which are unique as they require the cooperation of individuals in the community and are voluntary, where individuals choose to engage in behaviours [5, 6].

Even with increasing success from interventions aimed at reducing levels of pollution, behavioural interventions remain a necessary part of the strategy to reduce individual exposures to pollution [6]. Successful behavioural interventions require behavioural alterations by the public to reduce individual exposure to air pollution such as: staying indoors, wearing an appropriate mask, modification of physical activity, reducing the time spent outdoors, avoiding traffic-heavy areas and travelling at non-peak times [4–8].

The UK Health Security Agency (UKHSA) emphasised the need to increase awareness to enact behavioural change, enhancing the understanding of the problem in the population and providing clear advice on solutions [4]. Increasing awareness and providing clear advice are forms of Behaviour Change Communication (BCC) strategies where informative messages, often paired with an enabling environment, are used to persuade and maintain positive behaviour change [9, 10]. The majority of studies on BCC are focused on behaviours such as smoking cessation, dieting and physical activity [11], with only one review thus far being done on air pollution by Riley et al. The review found potential for behaviour change with communication, with avoidance behaviours showing the strongest evidence of change [5].

### Current communication approaches

Recent technological breakthroughs have resulted in air pollution monitoring and forecasting being widely available to the public [4, 7, 12, 13]. UK-AIR provides information on air pollution forecasts, pollution summaries, historical data, monitoring data and air pollution research [13]. Current pathways of information communication are general, including web pages, email bulletins, text messages (e.g., AiR TEXT) and social media. Agencies such as DEFRA, and London Air Quality Network (LAQN) provide advice to avoid air pollution on their websites [14, 15].

These current one-way communication approaches [13], however, which rely on the passive distribution of information, have been proven ineffective in forging an understanding within audiences and inspiring behaviour change [5]. Studies have shown that vulnerable populations are still not effectively receiving information [16] due to an incompatibility between information needed and wanted and information offered. Different studies found different air pollution communication needs, often dependent on context [17, 18]. To facilitate behaviour change, we should look at how actionable, understandable and relatable health information can be passed down from credible sources to communities [19].

### Novel approaches in communication: trusted information sources

Key aspects of effective communication revolve around the information sources (who is responsible for communicating the data?), the quality of the information been communicated, and the extent of reach (how can we reach vulnerable populations with the information?) [5, 16]. Environmental health risk communication effectiveness is maximized when information tailored to the needs of the audience is conveyed from a trusted source in a timely, consistent and easy-to-comprehend manner [20]. A review by UKHSA suggested the use of personalised messaging systems, and clearer information communication through trusted messengers to facilitate behavioural change [4], introducing the concept of information from a “trusted source”.

Having a trusted source relaying information renders the advice more acceptable to the public [5, 16, 20, 21]. Several studies have highlighted the concept of “trusted information sources”, such as doctors and other community health workers (from now referred to as community health professionals) who act as valuable information sources for families, children and patients [5, 20, 22]. Communication of air pollution information through community health professionals is better received compared to information directly from local or central government [5, 9]. Community health professionals have access to vulnerable members of the community, making them well placed to provide advice [21, 23]. A pilot project in 2020 focused on mobilising Health Care Professionals (HCPs) on air pollution explored this concept by providing training for HCPs to involve air quality advice to patients [21]. The project saw community benefits to training HCPs on air pollution and suggested that air pollution information should be communicated at all opportunities in community pathways, showing the potential of involving community health professionals in air pollution communication. Apart from this one project however, the integration of community health professionals in the communication pathway remains underexplored.

Previous studies on air pollution information communication have focused heavily on the improvement of information messaging, though a UK-based study by D’Antoni et al. (2019) showed that message format had minimal influence on advice adherence [10]. Thus, this study approaches communication in a novel way by focussing on the “information source” aspect of communication.

We aim to explore how community health professionals can be empowered to communicate air pollution information and advice from research agencies (e.g., DEFRA and London Air Quality Network (LAQN) [13, 15]) and local councils to the wider community, as a way to encourage the uptake of protective and avoidance behaviours in the population.

## Methods

### Theoretical framework

Kanter’s Structural Empowerment Theory (SET) suggests that the “role of management is to provide employees with the power tools that empower them to maximize their ability to accomplish their work in a meaningful way” [24]. This theory has been applied to a nursing context creating work conditions which allow nurses to provide the best care for patients [24, 25]. The theory identifies four structural conditions individuals need access to for workplace empowerment: information, support, resources, and opportunities [24, 25]. These structures are then distributed through formal power and informal power systems [25]. A 2002 study by Laschinger expanded the SET into the “nurse-patient empowerment framework” by including Spreitzer’s Psychological Empowerment Theory (PET) defined as “a psychological state which employees must experience for the success of empowerment strategies.” [26]. This concept comprises components of meaning, confidence, autonomy, and impact. These theories have been used only to limited extents in public health contexts outside nursing [24].

This research explores the application of these theories in relation to community health professionals who provide care to patients or residents. A 2016 paper highlighted the need for Health Professionals to be trained not just as experts, but as health change agents for the wider population [27]. Other studies reinforce this, suggesting health workers and nurses are well-placed to become such agents [28–30]. Thus, empowering these community health professionals could be a step toward social and health equity [29], leveraging their roles in the community to promote positive health behaviours.

### Study design

This study follows an interpretivism approach employing FGDs, “group interviews which capitalise on communication between research participants to generate data” [31]. FGDs encourage participation from those who might be less responsive in individual interviews, giving them opportunities to engage and challenge other participants. To better contextualise the findings from the FGDs, we asked participants for their gender and occupation. The FGDs were interactive and activity based, employing free-listing methods while asking respondents for their opinions of photographs shown (Additional file 1). All FGDs were conducted online with the online software, Microsoft Teams. A topic guide (Additional file 2) was used to facilitate the flow of discussion and ensure research objectives were met. During the question development process, a variety of open-ended question types were used. The questions began general and narrowed down to specific as FGDs went on. The study included a total of four FGDs, all conducted between May and July 2022.

### Participants and recruitment

This study is based in an inner London borough which meets most government National Air Quality Objectives but is some way from achieving 2021 WHO Guideline Values.

Recruitment was conducted through the local authority, which reached out to pre-existing groups with potential participants via email.

The study employed purposeful sampling, fitting for specific stakeholders rather than the general population. Inclusion criteria encompassed individuals capable of providing consent, English speakers, and two distinct groups: Healthcare Professionals (HCPs) with a healthcare background (e.g., General Practitioners, Community Health Workers), and other community leaders, such as social workers or group leaders, acknowledged and respected within the community.

Recruitment centred around the following groups: *Community Health and Well-Being Workers (CHWWs*), CHWWs are a new initiative in London and a first in the UK, although they are present in other countries such as the U.S. Their primary role is to work with individuals in their community to “identify and address health and wellbeing needs, improve health, prevent ill-health and reduce inequalities.” [32]. CHWWs hold visits to the houses of residents and signpost members of the community to relevant health resources. Previous studies highlighted the role of Community Health Workers (CHWs) in the U.S as agents of social change with an in-depth understanding of health and social care systems [29].

#### Healthcare Care Professionals (HCPs)

are often clinically trained and attached to a General Practitioner practice or a hospital in the area. Two of the participants in the group were Health Coaches, who provide services to support residents to adopt healthier lifestyles (e.g. weight loss or exercise programmes).

#### Social Prescribing Link Workers (SPLW)

play a pivotal role in linking individuals to local community activities and services with the aim of enhancing their health and overall well-being. A key aspect of this role involves establishing meaningful relationships by actively listening to the priorities and motivations of the individuals being assisted. SPLW identify factors influencing someone’s health and well-being and work collaboratively to explore and recommend services and activities that provide practical, social, and emotional support for an improved overall quality of life [33]. 

All participants were in occupations which carry the potential to act as agents of change in the community. The study was approved by the Imperial College Research Ethics Committee (ICREC), Reference Number 22IC7770.

### Data analysis

Online FGDs discussions were recorded and transcribed with the chat history being saved. Qualitative data was then analysed using a content analysis framework following four stages for the content analysis process: Decontextualization, Recontextualization, Categorisation and Compilation Bengtsson (2016). An interaction analysis was also carried out to add dimension, details and context to the content analysis [34]. The qualitative data underwent a meticulous manual analysis process. This involved a thorough examination and interpretation of the data, carefully reviewing and coding the information to extract meaningful insights.

Following the content analysis, this paper refers to several concepts as a guide to organising and interpreting the primary data collected, mainly the SET and PET while still remaining open to the data collected and themes outside of mentioned theories.

## Results

Four FGDs were conducted for the study, with a total of 19 participants (Table 1).

**Table 1:** Characteristics of FGD participants

Participant ID	Occupation	Gender
Focus Group 1: Community Health and Well-Being Workers (CHWW)		
A1	Community Health and Well-Being Worker	Female
A2	Community Health and Well-Being Worker	Female
A3	Community Health and Well-Being Worker	Female
Focus Group 2: Healthcare Care Professionals (HCPs)		
B1	Paediatric emergency registrar	Female
B2	Health Coach	Female
B3	Healthcare Assistant	Female
B4	Health Coach	Female
B5*	Academic GP trainee	Female
B5 was involved asynchronously after the initial FGD		
Focus Group 3: Social Prescribing Link Workers (SPLW) – Team 1		
C1	Senior SPLW	Female
C2	SPLW	Female
C3	SPLW	Female
C4	Senior SPLW	Male
C5	SPLW	Female
C6	SPLW	Female
Focus Group 4: Social Prescribing Link Workers (SPLW) – Team 2		
D1	A mix of non-clinical and clinical staff including SPLW, Case Managers and Clinical leads.	Female
D2		Female
D3		Female
D4		Female
D5		Female

The codes produced in the first two stages were sorted into sub-categories and larger categories in stages three and four (Categorisation and Compilation) of the content analysis. A coding table was then formed, where five main categories relevant to the research questions were identified, each encompassing diverse subcategories (Additional file 3).

The following section provides an overview of the current knowledge level and behaviour of the community professionals, contextualizing the findings of the study. We then describe in more detail the findings.

### Air pollution knowledge

Almost all of the participants highlighted that their level of air pollution knowledge was overall low, stating they should “learn more about it” (A1) A highly recognized point across all FGDs, was that London had poor air quality, with D2 commenting that “London, air pollution is huge as compared to rural areas”. High pollution hotspots can be found within inner London boroughs, which have “major roads which run through it” (D3). This shows a general awareness of the situation on both at a larger geographical and local level. Despite this, air pollution was mentioned as something which was rarely thought about in daily life. “It’s not something we think on a regular basis we need to stop and think about it.” (A1). The comments show a certain form of desensitisation to air pollution in London.

C1: It’s a given that people who have lived in London for so long, they just don’t think about it.

The health impacts of air pollution were similarly mentioned across all FGDs, with emphasis on how it disproportionately impacts vulnerable populations such as those suffering from Chronic Obstructive Pulmonary Disease (COPD), asthma and other respiratory diseases. The link between air pollution and health was often quickly and clearly drawn by the community health professionals, though they mentioned being unclear of specific percentages and facts. There was a consensus that air pollution was detrimental to health and thus of concern to those providing health advice.

Despite knowledge of health impacts, the majority of the participants admitted that they rarely spoke to or advised their clients on air pollution due to barriers outlined in later sections.

### Structural resources and support

This category, based on the “access to resources” and “assess to support” components of the SET, encompasses physical and social resources which influence community health professionals’ abilities to provide information and advice to the general community. Support includes other related sources that “maximize the efficiency of their role” [25].

### Time constraints

HCPs mentioned that hectic schedules made air pollution less of a priority topic in clinics. There was inadequate time in their everyday job scopes to perform this task and look up resources to bridge their knowledge gaps. It was mentioned that small physical collateral such as “resources which can be handed out to clients, even when they don’t have time to talk to them about it” (B2), would thus be helpful.

### Lack of supporting services

Lack of air pollution supporting services was mentioned by both SPLW FGDs, as part of their role was to signpost patients to related services for their health and social problems. The lack of air pollution support services made it difficult for them to integrate air pollution-related topics into their job scope.

A participant mentioned that “GPs have more information about the services that are available to patients” (D3), and how referrals to SPLWs from GPs could be accompanied with information on patient engagement with air pollution services. This shows the integrated care system in place and how different community health professionals can work with each other to achieve positive outcomes for the patients in their care. Currently, however, with a lack of known services which provide support for air pollution-related topics, SPLWs find it difficult to carry out their roles of signposting. The lack of services was not mentioned by HCPs or CHWW, possibly as there might be less emphasis on directing patients to relevant services in their role.

C1: I’m not sure what resources are already there in our local community, and some of the services that are available for residents at the moment, how can we have discussions with patients and signpost them to the right services?

### Physical collaterals for engagement

All FGDs brought up the need for collaterals on air pollution, be it resources which give them information about air pollution, or resources which can be passed on to residents or patients themselves. All FGDs, with exception of the CHWWs, asked the facilitator for advice on where they might find air pollution resources, which allowed for a clear segue into the section where they were shown examples of resources. Following this, participants requested that the links be sent to them for further reference. Overall, this behaviour reflects an interest in air pollution resources, but a lack of exposure to them.

It was mentioned that the “vast amount of information could be intense for residents” (C1) searching for information, and simpler methods of communication would be preferable. A commonly mentioned example was physical leaflets which could be distributed to residents with which had advice on them and further resources they could explore if they were interested in the topic. Other suggestions included pollution notices detailing the level of pollution in the area (D3), “Pollution alerts which operate like weather alerts, so professionals are aware of when pollution is heavy and advice more necessary” (A1) and “traffic light systems in communities which show a visual representation of pollution levels to residents” (D1). These are examples of resources which could be provided to community health professionals and the wider community.

C1: We can start with a leaflet with simple guidelines, but there could be websites and things so if people wanted to look more into it, you know, data statistics, all of that.

### Structural knowledge

This category, based on the “access to information” component of the structural empowerment theory [24], relates to factors influencing the knowledge and skills of community health professionals.

### Lack of knowledge

Lack of air pollution knowledge is a theme mentioned in the previous section and is the largest barrier to advising among all the professions. B5, a GP in training, acknowledged that while most healthcare professionals might possess basic knowledge and awareness of air pollution, they lacked sufficient depth to provide specific advice to patients. This point is reinforced by the other healthcare professionals, who displayed hesitancy and fear in giving “poor advice” (B2), and the need for a “strong evidence base” in the advice they are prescribing to the patients. This lack of knowledge contributes to the lack of advice being given out.

With current levels of knowledge, health coaches and SPLWs felt unqualified to provide advice on or speak to their patients about air quality. Participants from all roles agreed that air pollution is something they currently do not intentionally bring up with their patients due to their own limited understanding of it. All of the participants agreed that the lack of knowledge was a product of the lack of training or resources to advise on air pollution. Air pollution was not covered in detail in the curriculums for SPLWs and CHWWs training and is not part of the formal curriculum in medical school (B5).

### Advice to be distributed

Community health professionals did not only require knowledge in the form of “facts, but also advice to get the attention of residents” (A2). It was highlighted that there was not clear understanding about what advice should be given to residents. Table 2 shows some of the ‘only simple advice’, participants felt they could mention to residents.

**Table 2:** Behaviours Suggested by Participants over FGDs

Participant(s)	Behaviours suggested
A1	Turning off Air Fresheners
A1, B1, C1	Wearing Masks
B5, D4, C2	Staying Indoors
B5, C2	Purchasing Air Filter
C1	Breathing Exercises
B1	Taking Public Transport/Cycling

Participants mentioned a diversity of advice that they could give, with heterogeneity across and within their occupations. This likely stems from the lack of structured instructions or knowledge on what advice can and should be given, resulting in a lack of consistency on what they see as protective actions. There is a lack of knowledge on what would be considered “good advice” (B2) which should be distributed.

All FGDs highlighted that advice should be tailored to the participants they are speaking to as there is a need to “think of who you were really communicating it with” (B2) since different populations require different advice. High-risk groups such as children, the elderly, and patients who suffer from COPD and asthma, for instance, would require different advice compared to the general population. B1, a paediatrician, mentioned it will be difficult to convince parents to discourage their children from playing outside. Moreover, HCPs raised the concern that indoor air pollution is equally harmful as outdoor air pollution. B5 reiterated that “telling patients to stay indoors is not the best thing. If the air quality in their own home is really poor”. They suggested a form of risk stratification and clearer information on what advice should be given to people of different risk groups at different times.

B2: It would have to be thinking of who you were really communicating it with. You know that they would be the people who really are at high risk.

B1 (In Response): Yes, it’s like risk-profiling, is it specific enough at the moment, is there a strong enough evidence base to say [the advice] is definitely beneficial.

With more information on air pollution and the services which are available for resident use in the local community, community leaders would feel more comfortable speaking about it to their patients.

### Confidence as advisor

Even with sufficient knowledge, however, community health professionals could remain hesitant to advise due to a lack of psychological empowerment [26]. Thus, the next category touches on factors which influence the professional’s confidence in giving advice, relating to their personal perception of their skills and emotional capacity to do so.

### Difficulty in approaching topic

Participants of all occupations found that they lacked the skills to bring up air pollution with their patients and a formal way of including air pollution in health-related conversations they were having with their patients. CHWWs mentioned that the focus of their conversations was not solely on health but on current problems pertaining to each individual, making air pollution even more challenging to bring up.

After being shown current air pollution resources, the HCPs took an interest in the advice suggested on DEFRA’s website, highlighting that it largely suggested staying indoors. They discussed the “lack of positive framing of advice” (B1), stating that they find it difficult in their capacity to tell patients to refrain from doing something.

Some participants did not know how to approach conversations due to the large, invisible problem that is air pollution, which elicited a sense of futility amongst them. This would be damaging to their confidence to advise on the topics, as they felt as individuals, that they lacked power to effect significant changes in the issues. A5 highlighted that air pollution in London is out of individual control. This view on air pollution can demotivate people to talk about it, as attempts seem futile without proper suggestions for actionable change.

### Conflicting information

The existence of conflicting information makes community health professionals hesitant to provide advice, negatively impacting their self-assurance. This tension makes it difficult for them to be confident in the information they advise. The HCP FGD was the first to bring up existing tensions between the advice of staying indoors and the advice to go outside, which has other public health benefits. There should not be “more barrier for going outside, since there are negative health outcomes to NOT going outside” B1. The health coaches in the Focus group expressed concerns about sending conflicting messages after encouraging patients to go outside for physical exercise and mental wellness. SPLWs also touched on how the routines of going outside could be crucial to the mental health of the vulnerable population they work with, “going to the local shop is the one thing that they do in the day or the week” C1.

A5, however, highlighted that the suggestion to stay inside was for a short period, usually isolated days, and does not equate to asking patients to reduce physical activity. It could remain especially important for people who suffer from co-morbidities but are currently spending more time exercising outside, for instance, to be taken into consideration as they represent a type of intersectionality in this tension. They can be advised to safely continue their healthy habits while protecting themselves from air pollution during bad pollution episodes.

Given that these community advisors are not in constant contact with patients, this brings up an important point on how the advice should be given and how it needs to be emphasised that patients reduce activity in times of higher pollution, and not simply in general. Thus, recommendations to stay indoors perhaps lacked nuance and the advice given to different groups should be considered more deeply, as discussed later.

### Resource and skill building

Skill building is key to building the confidence of the advisors. It was previously highlighted that participants did not have training and resources, but they were quick to say that proper training and a good resource bank would be the largest factor which enabled them to provide advice. Overall, participants came to a consensus that training, alongside a resource bank, would be a definite facilitator to more actions, with many of their recommendations being centred around their resource and training needs. Suggestions included getting experts on the topic to facilitate sessions.

### Responsibility as advisors

This category relates to how the community health professional saw their responsibilities and duty to the residents in the community and their self-perception as a source of advice for the people they interact with. This relates to the possible meaning they attach to their job, an element of psychological empowerment.

### Expectation to advise

As community health professionals, all FGDs brought up how it was part of their role to provide advice and to help residents. HCPs spoke of the “power and that responsibility” (B1) attached to the role, as patients take note of what they are saying, a point that the whole FGD agreed with.

For CHWWs and SPLWs, they mentioned that it was part of their role to provide advice and solve problems for the residents they come into contact with. While they are not able to provide clinical advice like HCPs can, they saw it as meaningful to be able to speak to their residents about it, to “educate residents and help them better take care of themselves”, a point mentioned in FGD 3 which the group agreed with.SPLWs saw providing air pollution advice as a way they can “work towards supporting our COPD patients” (D3) and expressed curiosity in approaching it to “empower residents to see what’s best for them” (D3). FGD 4 highlighted the need to engage with patients on this topic as well, to gain a better understanding of the support required. Overall, participants saw the inclusion of air pollution-related topics as relevant to their roles and saw a possibility in providing advice.

### Trust and connection

HCPs in particular expressed awareness of the weight of their advice to their patients and identified as trusted sources. The concept of a ladder of trust was mentioned, where nurses and doctors were seen as the most trustworthy sources. While not explicitly mentioned by CHWW and SPLWs, their descriptions of their job scopes alluded to a high amount of interaction with the people they are working with. CHWWs often needing to conduct home visits and aid residents in a continual process of “problem-solving” (A1), and SPLWs “promoting self-care and independence” (C1).

### Receptiveness to advice

While earlier categories centre around the empowerment of community health professionals, this theme focuses more on how advice is received by the community. It includes factors which either increase or lower the likelihood of the audience following advice from the community health professionals.

### Personal risk level

The advice given by these community health professionals is “helpful to residents who want to listen” (A1), it thus remains important to be aware of these factors. Participants felt that patients were more likely to listen if they suffered from co-morbidities such as COPD and asthma, where pollution would clearly inhibit their quality of life. These patients likely are more conscious of pollution in the first place. A5 mentioned that they had patients who actively sought advice for air pollution due to their own interests. At the same time, other residents in what those might consider “low risk” groups are unlikely to listen to advice, a sentiment shared by both HCPs and SPLWs.

### Hierarchy of needs

Audiences are less likely to listen and act on advice as they see air pollution as a non-priority in their lives. This was a point more strongly mentioned in FDGs involving CHWW and SPLWs rather than HCPs, possibly due to the more interdisciplinary, integrated nature of the role where the focus is not solely on healthcare. “Air pollution was not a priority for any of the patients they saw” (C4) and part of why they did not give advice was because patients were not asking for it. Other participants agree with this, stating that given the large plethora of issues they cover with patients, at times air pollution and its impacts could take the backseat. This is especially due to the desensitisation the patients feel towards air pollution, given that they’ve “lived in London for so long, they just don’t think about it” (C1).

CHWWs are in a unique position where they do not work on health problems alone, but often have to take into account the wider determinants of health. They expressed that at times it is difficult to even centre the conversations they have on health, with residents being more focused on what they perceive as time-sensitive issues, be it a leak in the house or other personal issues. This limits the receptiveness residents have to the advice, and sheds insight on possible reasons for disinterest in air pollution amongst residents. It also suggests difficulties CHWWs might have in broaching the topic given the diversity of issues they are to discuss.

### Ability to engage in advice

It was also suggested that audiences are more likely to respond if the advice is easily accessible to them. FGD 3 found felt that residents would face issues trying to assess current resources given the vast quantity of resources which can be overwhelming, they suggested that small specific steps would be better received by their target group, making them more likely to follow the advice. Advice should also be more specific and “prescriptive than terms like reducing activity” (B5). It would be beneficial to include specific timeframes they should stay inside, or specific pollution bandings they should avoid going outside.

At the same time, the advice given must be feasible for the target groups. For instance, wearing a mask would likely be easy advice given the common usage during the COVID pandemic (C1), but telling a cyclist not to cycle because of air pollution might not go over as well (D2). HCPs also saw the need to provide alternatives when telling patients not to do something. For example, when they are told not to go outside due to air pollution, they should be offered alternatives such as online exercise classes (B2). Local level policies could support this by for instance opening community spaces on pollution-heavy days, so children have an alternative to spending time outside (B1). This would empower the population to follow the advice and increase adherence.

The concept of financial feasibility was mentioned on two occasions, once by a HCP and the other by a SPLW. In both instances, the purchasing of an air filter for the household was used as an example. They mentioned the purchasing of the filter as a good preventive action to take but acknowledged this was not financially feasible for all residents. This was a unique code as it introduced more dimensions to the issues at hand and reflected how other social determinants of health such as financial status could determine how residents acted on the advice given.

**Fig. 1:**
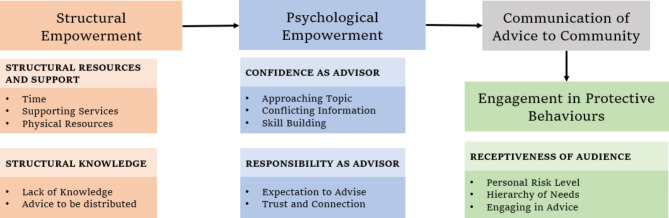
Application of Categories to Empowerment Models

In summary, we identified current barriers to empowerment of community health professionals in the borough, and recommendations to overcome said barriers to achieving effective communication. SET and PET [24] were used as guiding frameworks, undercovering themes relevant to the local context (Fig. 1).

## Discussion

We found a low level of general knowledge on air pollution and engagement in air pollution communication amongst community health professionals in the locality, despite this, community professionals acknowledged the need for air pollution advice and communication to be integrated into their roles. Community leaders are not able to provide accurate information and advice if they lack the knowledge and resources to do so [35]. Thus, while community leaders are well-placed to facilitate better communication of information and advice, their potential is currently underutilized in current information dissemination pathways [5, 19, 22]. There should be deliberate efforts to equip community leaders with relevant skills and knowledge to provide advice to the community around them [36].

We identified key gaps which hinder the empowerment of community health professionals in current community structures. The needs for time, physical materials and support services mirror those identified in previous studies on Nurse Empowerment [24, 26]. The specifics of these resources, however, are highly contextualized.

We also identified a lack of structural knowledge within community health professionals. Knowledge is not a component in the SEM, but overlaps with “access to information”, which involves the sharing of information. There are currently resources compiled by other organizations for HCPs, including “Air Pollution Guide for Health Professionals” by the Greater London Authority [37] and Global Action Plans (GAP) Clean Air Hub, which compiles various resources for varied audiences [38]. None of the participants had an awareness of these, but commented that they would be very useful, showing that these resources are not utilized. Organizations should have systems which provide community health professionals with adequate knowledge on air pollution and air pollution advice or exposes them to current resources available. The need to include information on air pollution in medical curriculum was also highlighted by WHO during an expert consultation [39], reflecting how this is a significant knowledge gap.

Participants lacked confidence to provide advice on air pollution, mostly due to a lack of training and skill-building. They found difficulties in approaching the topic brought about by a sense of futility regarding individual action, a psychological factor mentioned by non-community health professional individuals in previous studies [5, 9]. They also felt hesitant to advise due to conflicting information. The WHO highlighted the need for a standardized approach to communication of risks and advice to patients, as conflicting messages from medical professionals result in misconceptions about air pollution [39], showing the significance of this gap.

Their perceived responsibility to advise acts as a psychological enabler, motivating community health professionals to provide advice. The attachment of a sense of duty to the people they interact with provides meaning to their roles, encouraging them to want to learn more to give high-quality advice, as displayed by their eagerness to learn and obtain materials from the FGDs. While this can be loosely related to the “meaning” component of PET, it was not explicitly covered in any previous studies, showing it could be a new concept for consideration.

Factors related to behaviour change concern the target audience receiving advice. This is especially relevant in this study, where the purpose of effective communication is to promote positive behaviour change [40]. Their ability to receive and act on the advice is determined by factors such as their risk level, ability to engage in advice and their hierarchy of needs. Previous studies have also shown that people who are part of at-risk groups such as individuals with asthma and the elderly are more likely to engage in air pollution avoidance behaviours than people who self-perceive as healthy [5, 9, 16]. Other barriers, such as technology and language [16], were mentioned in the literature, but not in the course of the study. The FGDs highlighted that instructions should be clear and simple to allow residents to comprehend the advice instructions, a point brought up in previous studies [9].

The content of advice to be given to audiences was discussed in detail, with community health professionals making suggestions based on their understandings of what audiences might find effective. The characteristics to be included in the advice are highlighted below.

Firstly, stratification of groups to advise. Groups should be differentiated according to demographics and level of risk. Those who are more heavily impacted by air pollution would need advice more often or on days with lower pollution thresholds. Risk stratification is widely employed in existing air pollution communication channels, and is a form of tailoring information [16, 20]. However, there have been critiques that an overfocus on at-risk groups, leads to a lack of attention on the general risk of air pollution [16]. From the perspective of community health professionals, however, risk-stratification allows them to broach the topic with audiences who are most likely to respond while speaking to other residents about other topics of importance, which is ultimately the purpose of their roles.

Next, the provision of alternatives to outdoor activity. When advice includes avoidance actions such as staying indoors, community health professionals should pair it with alternatives to outdoor activities, such as online exercise sessions or visits to community centres nearby for indoor activities. Riley et al.’s review (2021) suggested similarly, that giving alternative actions to be taken increases the chance of response to advice [19]. The FGDs, however, also mentioned low levels of knowledge on local actions or alternatives they could suggest.

Lastly, advice should be clear and specific about the actions it is suggesting. For instance, the usage of the terms “reduce activity outside” and “pollution is bad” are unclear, and participants suggested providing timeframes to stay indoors and citing specific numbers on the pollution index will be more specific. Advice also has to be easy to understand and follow for the residents, a point supported by previous findings [5]. Studies have shown a strong need for advice and information to be clear of overly technical language [16, 40]. At the same time, it cannot be overly simplified (too regional or national) such that people feel distant and disengaged [40]. Localised advice is likely the best way forward.

Recommendations therefore include both structural and psychological empowerment in organizational settings to provide an enabling environment for community health professionals to provide advice [24]. To achieve structural empowerment, recommendations should facilitate empowerment of professionals through the two power systems, “informal” and “formal” [41]. Organizational positions and job activities are the key sources of formal power, while informal power is derived from relationships within the organization [24]. Formal power can be increased through the training sessions, while informal power can be encouraged through increased interaction between community health professionals such as SPLWs and the referring GPs. These recommendations mirror those given by Public Health England (PHE) after their review in 2019, stating the need to guide the public through training HCPs, school governors and staff, and social care professionals [4].

### Limitations

The methodological approach adopted during this study explored the concept of trusted information sources to disseminate and personalize advice, which is a method to achieving effectiveness [39]. The study, however, had limitations, the first on the number and composition of FGDs. While it would have been ideal to have two FGDs of each stakeholder group (CHWW, HCPs and SPLWs), the tight timeline made it possible to only recruit one of each for CHWWs and HCPs. The minimum of three focus groups was enough to reach saturation across general themes for community health professionals, but the themes specific to each role could have been more apparent with more focus groups.

The homogenous sampling often yields results less generalizable to the larger stakeholder population [42], a notable result of the eventual population demographics was that participants were almost all female. Women are less likely to give advice compared to men for difficult questions [43]. and one’s gender at times can determine how the advice is received [44]. The predominance of female participants in the majority of the Focus Group Discussions (FGDs), could be attributed to the demographics of the specific groups targeted for the study, where there might be a higher proportion of females involved in healthcare and community roles. While gender was not explicitly mentioned through the FGDs, it is important to note that the data adopts largely female perspectives and that the participant’s gender shaped their social experiences and views regarding the subject.

## Conclusion

This exploratory research identified ways in which community health professionals such as CHWWs, HCPs and SPLWs can be empowered to provide advice on air pollution. Overall, there is potential for community health professionals to be integrated into air pollution communication strategies. The established connection and trust with the community make them well-placed to provide advice to the wider community, who view them as trusted information sources. To their understanding, the community is more likely to follow advice which is simple, specific, well-framed and tailored. Currently, however, community health professionals still lack the resources, knowledge, and skills to confidently engage the community on the topic. In light of the study’s findings, it is crucial to ensure that Community Health Professionals possess the knowledge and confidence to effectively advise patients on air pollution. Structured training programs, ongoing educational resources, and confidence-building initiatives should be implemented to address identified barriers, such as a lack of resources and time constraints. Establishing a clear responsibility for community health professionals in advising on air pollution, integrating tailored communication training, and fostering collaboration with environmental experts can enhance their ability to provide informed advice.

While this study is exploratory in nature, it lays the groundwork for future research aimed at designing interventions where the proposed model can be tested. Future research should also broaden its scope to encompass a diverse range of community health professionals such as link workers, school nurses, teachers, and faith leaders. This will give more insight into which community roles are most suited to provide advice on air pollution. The inclusion of more perspectives will allow for more tailored and specific training and skill-building for each job role, which allows for better structural empowerment.

Lastly, more research should be done with local communities, with a focus on understanding their needs for pollution communication, and barriers to accessing and using air pollution information. With more perspectives from residents, more effective communication strategy changes can be applied.

### Electronic supplementary material

Below is the link to the electronic supplementary material.

Supplementary Material 1

Supplementary Material 2

Supplementary Material 3

## Data Availability

The datasets used and/or analysed during the current study are available from the corresponding author on reasonable request.

## Abbreviations

BCC: Behaviour Change Communication
CHWW: Community Health and Wellbeing workers
COPD: Chronic Obstructive Pulmonary Disease
DEFRA: Department for Environment, Food and Rural Affairs
FGD: Focus Group Discussions
GAP: Global Action Plan
GP: General Practitioner
HCP: Healthcare Professional
SPLW: Social Prescribing Link Workers
PET: Psychological Empowerment Theory
PHE: Public Health England
SET: Structural Empowerment Theory
WHO: World Health Organization
