# Supplementary material for: Empowering community health professionals for effective air pollution information communication
Source: BMC Public Health. 2023 Dec 20;23:2547. doi: 10.1186/s12889-023-17462-1 (PMC10734129; doi:10.1186/s12889-023-17462-1)
Supplement: Supplementary file 1 — Supplementary Material 1 [file 12889_2023_17462_MOESM1_ESM.docx]

Additional file 1: Photographs used for interactive sections of FGD

a. Pictures used to show different types of air pollution communication resources

| **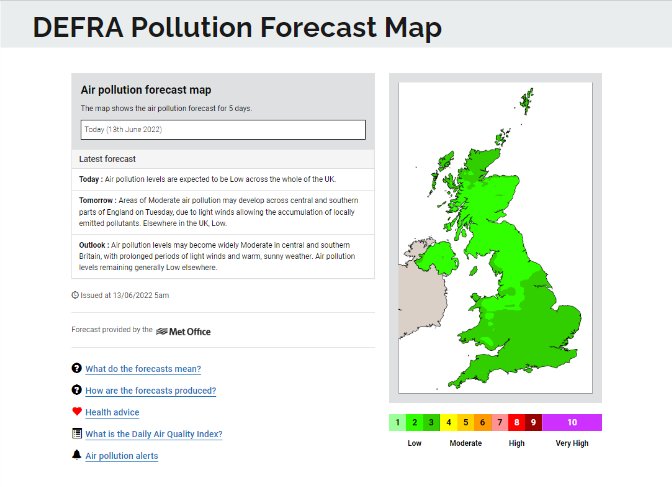** | **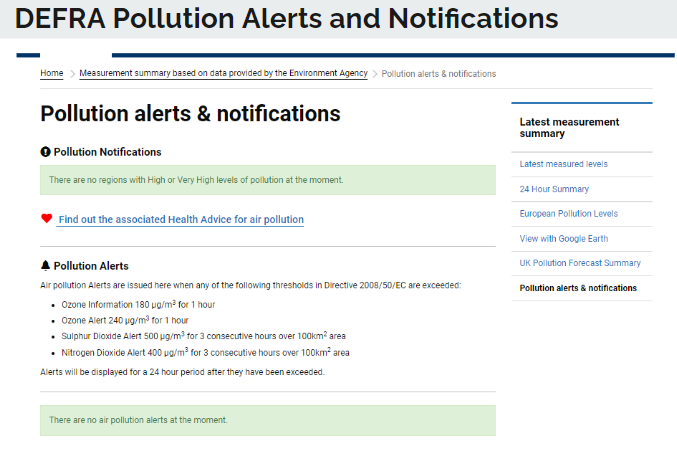** |
| --- | --- |
| **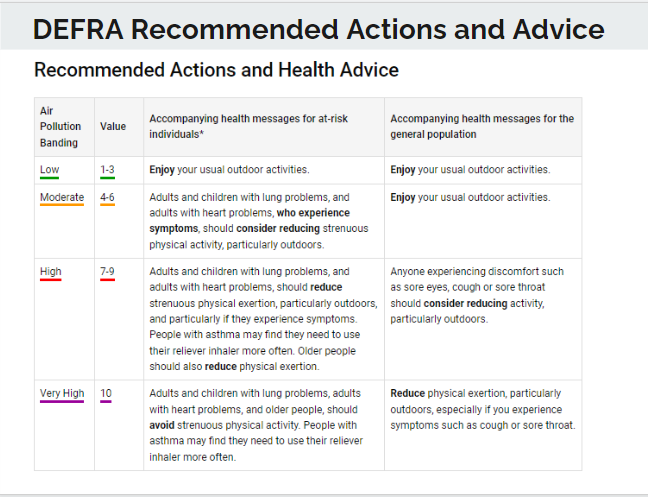** | **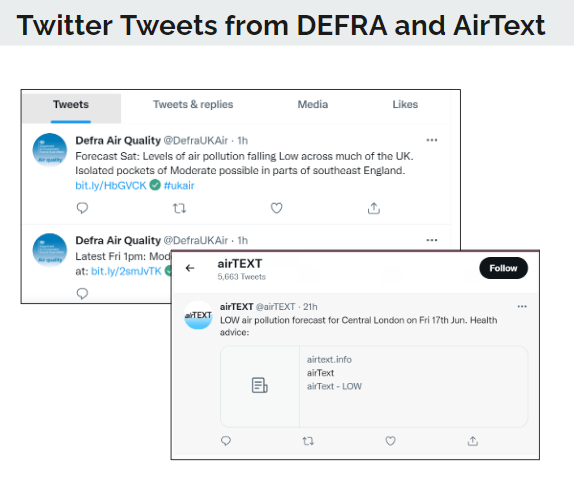** |
| **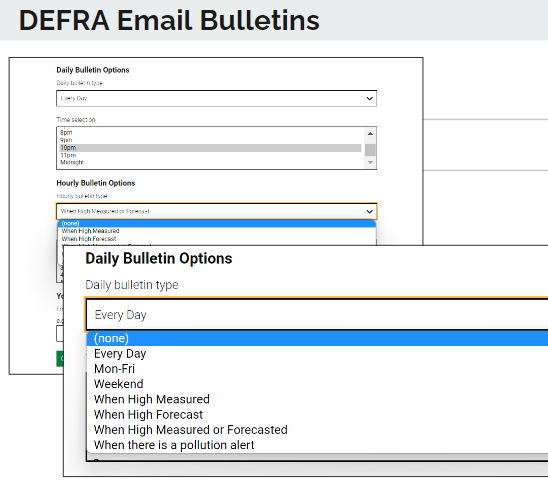** | **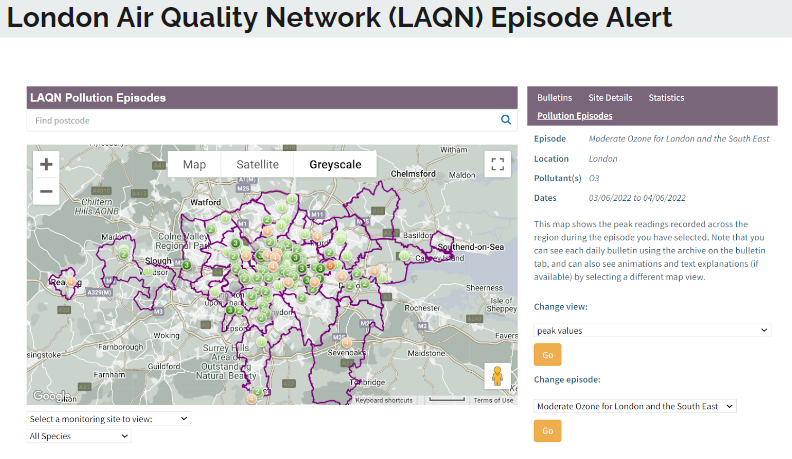** |
| **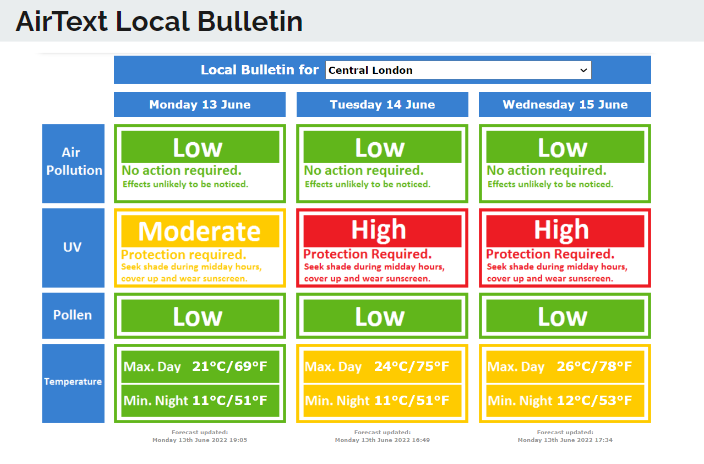** | **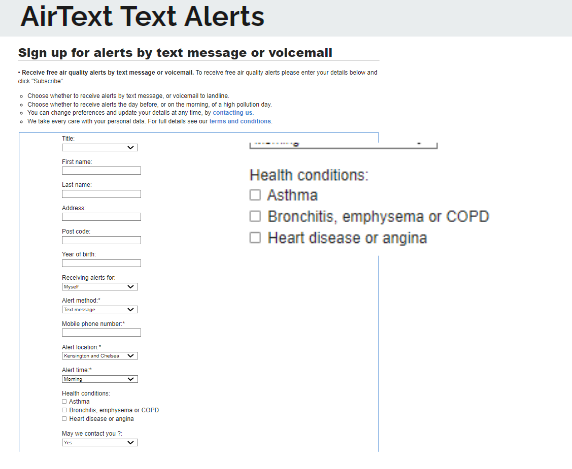** |
| **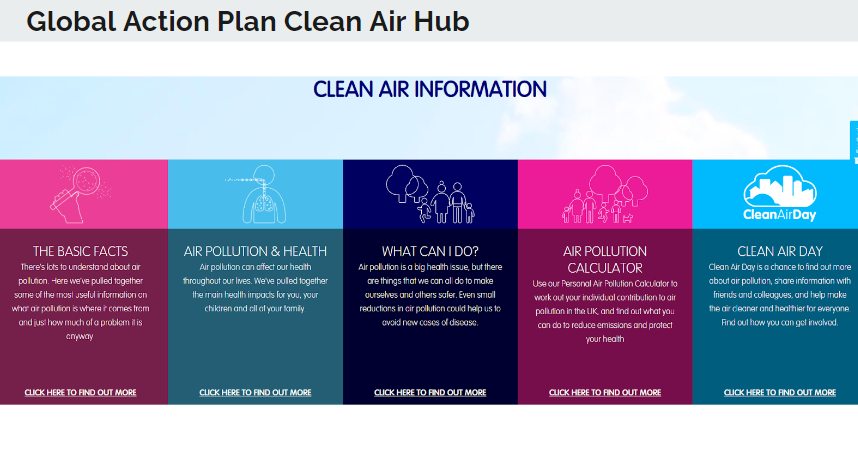** | **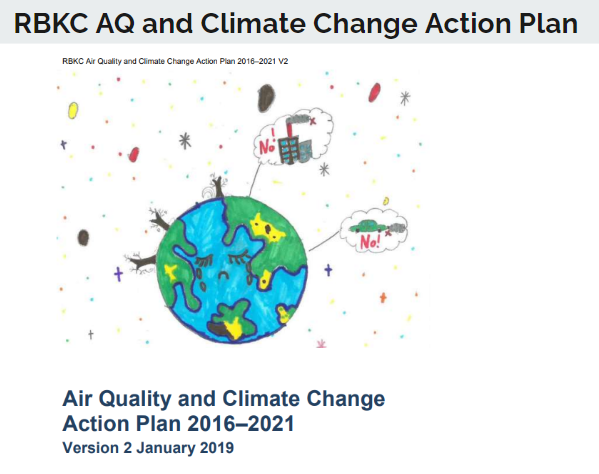** |

b. Pictures used to show resources currently available to teach HCP how to provide advice

Global Action Plan Clan Air Hub training video for talking to patients about air pollution (40 min) available at <https://www.cleanairhub.org.uk/home>
