# Supplementary material for: Empowering community health professionals for effective air pollution information communication
Source: BMC Public Health. 2023 Dec 20;23:2547. doi: 10.1186/s12889-023-17462-1 (PMC10734129; doi:10.1186/s12889-023-17462-1)
Supplement: Supplementary file 2 — Supplementary Material 2 [file 12889_2023_17462_MOESM2_ESM.docx]

Additional file 2: Topic Guide for Focus Group Discussions

| *Opening the Session: Gather general Information and Introduction to the FGD* |
| --- |
| *General Questions: Understanding their current beliefs and attitudes towards air pollution* |
| 1. What do you know about air pollution?   The similarities and differences brought up will be discussed in further detail. |
| - What do you know about the impacts of air pollution? |
| - What do you think can be done to reduce air pollution in general? |
|  |
| *Current Interaction with Air Pollution Information* |
| 1. Presentation of images of different types of air pollution information available (monitoring data, pollution episode warnings, general advice, air pollution policies) (Appendix 1a)   You have just been shown a variety of air pollution information resources… |
| 1. What are the current resources that you look at if any? |
| 1. Do you feel that these resources are helpful to you? |
|  |
| *Professional Roles and Role in Community* |
| As a Healthcare Professional/Teacher/School Nurse/Community Worker do you see it as part of your role to protect patients from air pollution? |
| What actions do you think you can take to protect patients from air pollution? |
| What is hindering you from taking these actions? |
|  |
| *Barriers and Facilitators to Air Pollution Information Communication* |
| 1. How can information be better communicated to you (as a Healthcare Professional/Teacher/School Nurse/Community Worker) to help you better respond to poor air quality? |
| 1. The following shows some pictures of collaterals for air pollution information for Health Professionals. (Appendix 2b)  - For Health Professionals: Have you seen this before? - For Others: Do you feel something similar to this collateral but for teachers/community leaders would be helpful? |
| 1. Conversely, what are some aspects which would make the air pollution information unhelpful for you? |
|  |
| *Conclusion* |
| The purpose of this study was to better understand how air pollution information can be communicated to healthcare professionals to enable them to communicate risk and protection to patients under their care….  How can we help you help the wider community? Please give us any concluding statements. |
