# Supplementary material for: Empowering community health professionals for effective air pollution information communication
Source: BMC Public Health. 2023 Dec 20;23:2547. doi: 10.1186/s12889-023-17462-1 (PMC10734129; doi:10.1186/s12889-023-17462-1)
Supplement: Supplementary file 3 — Supplementary Material 3 [file 12889_2023_17462_MOESM3_ESM.docx]

Additional file 3: Categories and subcategories identified through content analysis

| Category | Sub-Categories | Freq. in FGDs* | Codes |
| --- | --- | --- | --- |
| Structural Resources and Support | Time Constraints | HCP | Business in Role  Lack of Time |
|  | Lack of Supporting Services | SPLW 1,  SPLW 2 | Difficulty in Signposting  Supporting Services  Unsure of Council Actions |
|  | Physical Resources for Engagement | CHWW,  HCP,  SPLW 1,  SPLW 2 | Leaflets and flyers  Visual Reminders  Pollution Notices  Distribution to Patients |
| Structural Knowledge | Lack of Knowledge | CHWW,  HCP,  SPLW 1,  SPLW 2 | Need to Learn  Unsure of Advice  Lack of Resources  Lack of Training  Curriculum |
|  | Advice to be Distributed | CHWW,  HCP,  SPLW 1,  SPLW 2 | Well-structured  Simple to Follow  Financially Feasible  Clear  Risk Stratification  Tailored Advice  Based on Target Group  For Vulnerable Populations |
| Confidence as Advisor | Difficulty Approaching Topic | CHWW,  HCP,  SPLW 1,  SPLW 2 | Positive Framing  Include in Conversation  Inability to Change  Helplessness  Futility  Provision of Alternatives |
|  | Conflicting Information | HCP,  SPLW 1,  SPLW 2 | Physical Activity  Mental Health  Hesitancy |
|  | Skill Building | CHWW,  HCP,  SPLW 1,  SPLW 2 | Usefulness of Resource  Training Sessions  Asking for Resources After  Interest in Topic |
| Responsibility to Advise | Expectation to Advise | CHWW,  HCP,  SPLW 1,  SPLW 2 | Responsibility  Imparting Knowledge  Access to Risk Groups  Role in Job |
|  | Trust and Connection | CHWW,  HCP,  SPLW 1,  SPLW 2 | Hierarchy of Trust  Built Relationships  High Community Interaction  Empowerment  Problem Solving |
| Receptiveness to Advice | Hierarchy of Needs | CHWW,  SPLW 1,  SPLW 2 | Social Determinants  Financial Difficulties  Priorities |
|  | Personal Risk Level | CHWW,  HCP,  SPLW 1,  SPLW 2 | Personal Risk  Level of Awareness  Easy to Follow Advice  Desensitisation to Problem  Detachment from Health Issues |
|  | Ability to engage in Advice | HCP,  SPLW 1,  SPLW 2 | Financial Ability  Understanding of Advice |
